# Synthesis, crystal structure, spectroscopic features and Hirshfeld surfaces of 2-methyl-3-[(2-methyl­phen­yl)carbamo­yl]phenyl acetate

**DOI:** 10.1107/S2056989019000021

**Published:** 2019-03-05

**Authors:** Mavişe Yaman, Şukriye Cakmak, Necmi Dege, Mustafa Odabaşoğlu, Vadim A. Pavlenko, Halil Kutuk

**Affiliations:** a Ondokuz Mayıs University, Faculty of Arts and Sciences, Department of Physics, 55139, Samsun, Turkey; b Vocational School of Health Services, Environmental Health Programme, Sinop, University TR-57000, Sinop, Turkey; c Pamukkale University, Department of Chemistry and Chemical Processing Technologies, 20070 Kınıklı-Denizli, Turkey; d Taras Shevchenko National University of Kyiv, Department of Chemistry, 64, Vladimirska Str., Kiev 01601, Ukraine; e Ondokuz Mayıs University, Faculty of Arts and Sciences, Department of Chemistry, 55139, Samsun, Turkey

**Keywords:** crystal structure, amide, X-ray diffraction, Hirshfeld surface, hydrogen bonding

## Abstract

The title compound, C_17_H_17_NO_3_, was synthesized, characterized by IR spectroscopy and its crystal structure was determined from single-crystal diffraction data. The asymmetric unit contains two mol­ecules, which adopt different conformations. In one mol­ecule, the acet­oxy and the terminal 2-methyl­phenyl groups are positioned on opposite sides of the plane formed by the central benzene ring, whereas in the other mol­ecule they lie on the same side of this plane. In the crystal, the mol­ecules are linked through strong N—H⋯O hydrogen bonds into chains along [010]. Hirshfeld surface analysis and fingerprint plots were used to investigate the inter­molecular inter­actions in the solid state.

## Abstract 2

2-Methyl-3-[(2-methyl­phen­yl)carbamo­yl]phenyl acetate was synthesized, characterized by IR spectroscopy, and its crystal structure was determined from single-crystal data. In the crystal, mol­ecules are linked by N—H⋯O hydrogen bonds. The two independent mol­ecules in the asymmetric unit adopt different conformations.

## Chemical context   

Amides and their derivatives are extremely important biologically active compounds. Amide groups are present in a number of natural products, polymers and pharmaceuticals (Valeur & Bradley, 2009 ▸; Xiang *et al.*, 2012 ▸). Amide derivatives have been found to exhibit biological and pharmacological activities such as anti­tumor, anti­microbial, anti­bacterial, anti­fungal, anti-HSV, analgesic, anti-inflammatory and anti­cancer (Carbonnelle *et al.*, 2005 ▸). Moreover, amide-based compounds represent an important group of efficient chelating ligands (Strotmeyer *et al.*, 2003 ▸; Sliva *et al.*, 1997 ▸; Pavlishchuk *et al.*, 2011 ▸; Gumienna-Kontecka *et al.*, 2007 ▸). Recently, we synthesized and studied some new substituted secondary benzamide derivatives obtained as a result of the inter­action of aniline-based compounds with acyl chlorides (Çakmak *et al.*, 2016 ▸; Kırca *et al.*, 2018 ▸; Demir *et al.*, 2015 ▸; Kansız, Çakmak *et al.*, 2018 ▸). Among them, 3-acet­oxy-2-methyl-*N*-(4-meth­oxy­phen­yl) benzamide was found to exhibit good anti­oxidant activity (Demir *et al.*, 2015 ▸). As a continuation of this work, we prepared the title compound and studied its spectroscopic and structural features.

## Structural commentary   

**Figure 1:**
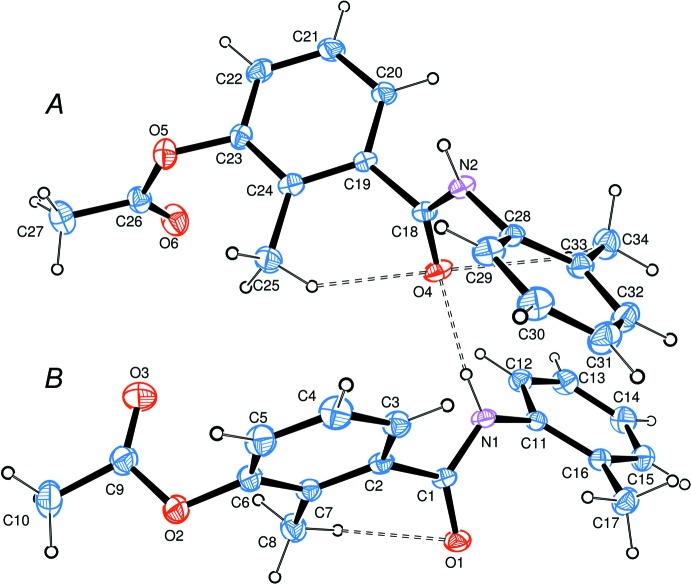
The asymmetric unit of the title compound, with displacement ellipsoids drawn at the 50% probability level.

The asymmetric unit of the title compound (Fig. 1 ▸) contains two mol­ecules, *A* and *B*, which adopt different conformations that can be characterized by the mutual arrangement of the acet­oxy and terminal 2-methyl­phenyl groups with respect to the plane of the central benzene ring: in mol­ecule *A* they lie on different sides of this plane, whereas in mol­ecule *B* they are positioned on the same side. The torsion angles characterizing the conformation details are summarized in Table 1 ▸. The dihedral angles subtended by the aromatic rings are 54.33 (12) and 66.68 (11)° in mol­ecules *A* and *B*, respectively. The mol­ecular conformations are stabilized by weak intra­molecular C—H⋯O contacts (Table 2 ▸). All bond lengths and angles are typical of similar compounds, bearing in mind the effect of inter­molecular hydrogen bonds on the geometry of the amido groups.
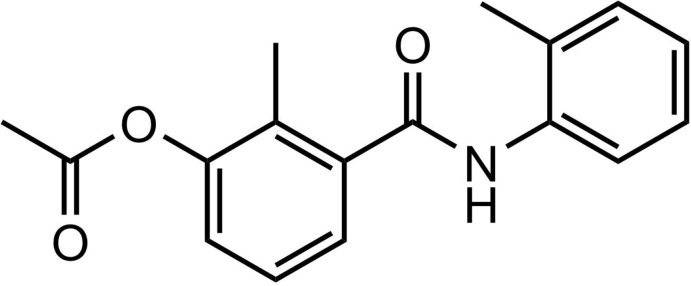


## Supra­molecular features   

**Figure 2:**
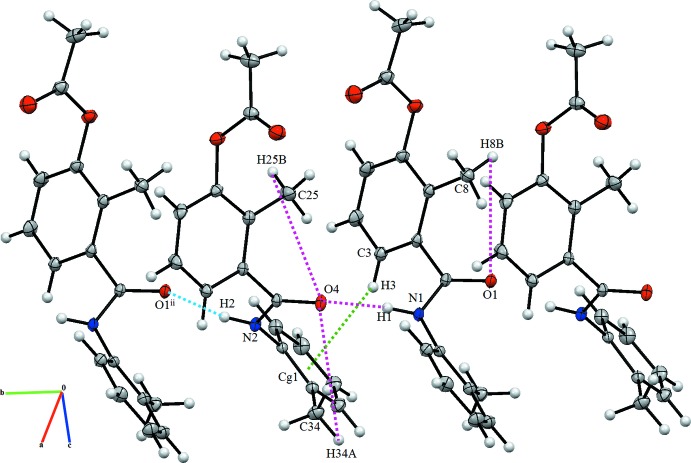
Packing diagram of the title compound showing the short inter­molecular contacts. *Cg*1 is the centroid of the C28–C33 benzene ring.

The packing diagram of the title compounds is presented in Fig. 2 ▸. In the crystal, the mol­ecules are linked through strong N—H⋯O hydrogen bonds (Table 2 ▸) into chains along [010]. They are further linked by C—H⋯O and C—H⋯π contacts (Table 2 ▸).

## Database survey   

A search in the Cambridge Structural Database (CSD version 5.39, update of August 2018; Groom *et al.*, 2016 ▸) for 3-acet­oxy-*N*-phenyl­benzamide derivatives gave three hits: 3-acet­oxy-2-methyl-*N*-(4-methyl­phen­yl)benzamide (HEJBIK; Kırca *et al.*, 2018 ▸), 3-acet­oxy-2-methyl-*N*-phenyl­benzamide and 3-acet­oxy-2-methyl-*N*-(4-meth­oxy­phen­yl)benzamide (HEJBOQ and JUMCEB, respectively; both Demir *et al.*, 2015 ▸). The structure of HEJBIK is especially close to that of the title compound: it also contains two mol­ecules in an asymmetric unit and is isostructural to the title compound with the exception of one methyl group (2-Me in the title compound and 4-Me in HEJBIK). The two independent mol­ecules in HEJBIK have different conformations in the same manner, as in the title structure. In the two structures HEJBOQ and JUMCEB, the acet­oxy groups and the terminal benzene rings are positioned on opposite sides of the planes formed by the central benzene rings. In all these structures, the mol­ecules are linked into chains by N—H⋯O hydrogen bonds.

## Hirshfeld surface analysis   

**Figure 3:**
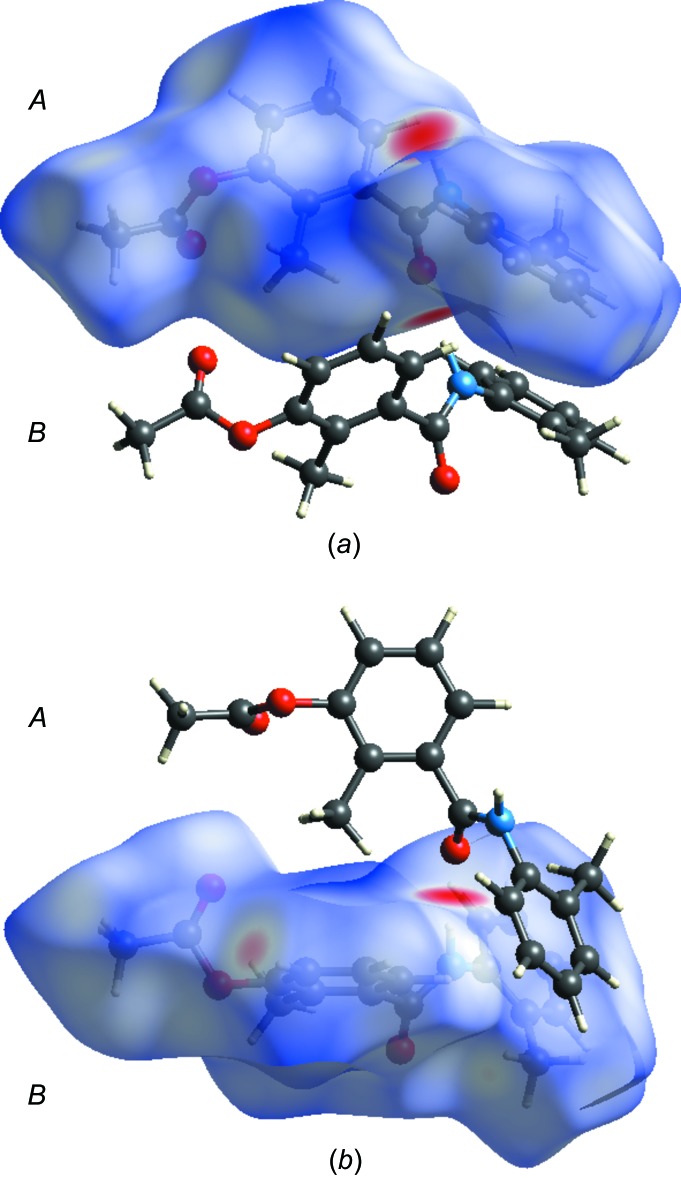
Hirsfeld surfaces of 3-acet­oxy-2-methyl-*N*-(3-methyl­phen­yl) benzamide (three-dimensional *d*_norm_ surface): (*a*) mol­ecule *A* and (*b*) mol­ecule *B*.

**Figure 4:**
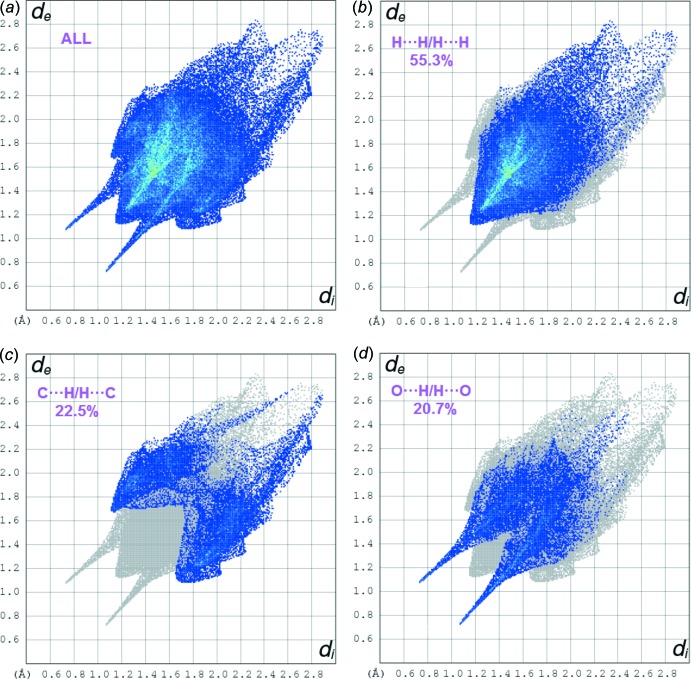
The fingerprint plots for mol­ecule *A*: (*a*) all atoms_inside_⋯all atoms_outside_ (100%), (*b*) H_inside_⋯H_outside_/H_outside_⋯H_inside_ (55.3%), (*c*) C_inside_⋯H_outside_/H_outside_⋯C_inside_ (22.5%) and (*d*) O_inside_⋯H_outside_/H_outside_⋯O_inside_ (20.7%).

**Figure 6:**
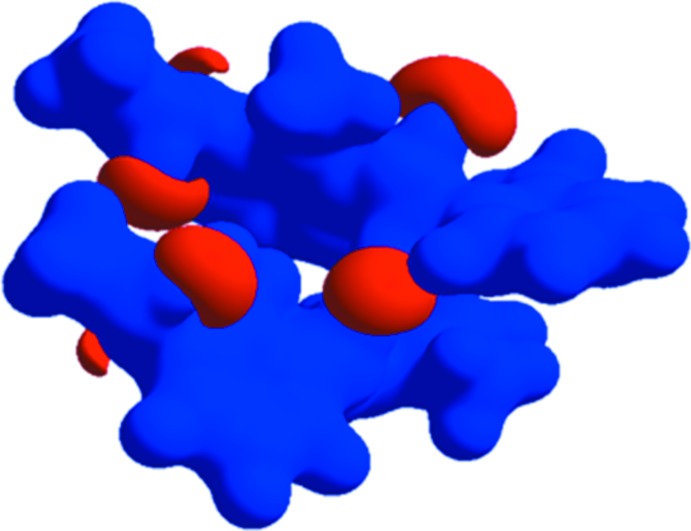
Electrostatic potential mapped on the Hirshfeld surface (± 0.25 a.u.).

The mol­ecular Hirshfeld surfaces (*d*_norm_) for mol­ecules *A* and *B* of the title compound generated using *CrystalExplorer3.1* (Wolff *et al.*, 2012 ▸) and are presented in Fig. 3 ▸. The *d*_norm_ values are mapped on the Hirshfeld surfaces using a red–blue–white colour scheme (Spackman & Jayatilaka, 2009 ▸) as follows: the dark-red spots indicate the closest contacts related to the N—H⋯O hydrogen bonds, the other short inter­molecular contacts appear as light-red spots, blue regions depict positive *d*_norm_ values, and in the white regions the lengths of the contacts are exactly equal to the sum of van der Waals radii (*d*_norm_ = 0). Analogous dark-red spots related to the N—H⋯O inter­actions were observed on the Hirshfeld surfaces of similar mol­ecules (Şen *et al.*, 2017 ▸; Gümüş *et al.*, 2018 ▸; Kansız & Dege, 2018 ▸). Figs. 4 ▸ and 5 ▸ show the two-dimensional fingerprint plots for mol­ecules *A* and *B*, respectively. For both mol­ecules, the contributions from the H⋯H/ H⋯H contacts are the largest (55.3 and 53.9% for *A* and B, respectively). The contributions of the other inter­molecular contacts are as follows: C⋯H/H⋯C (22.5%) and O⋯H/H⋯O (20.7%) for *A* and C⋯H/H⋯C (23.8%) and O⋯H/H⋯O (21.7%) for *B*. The Hirshfeld surface mapped over the electrostatic potential n (±0.25 a.u.) is shown in Fig. 6 ▸ where blue regions correspond to positive electrostatic potential and red spots related to the oxygen atoms represent the areas of negative electrostatic potential; the distribution is analogous to that in a similar compound (Yaman *et al.*, 2018 ▸).

## Vibrational spectrum   

**Figure 7:**
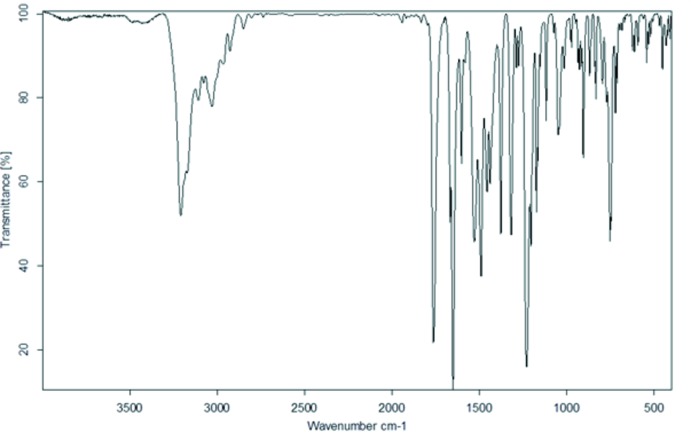
IR spectrum of the title compound.

The IR spectrum of the title compound (KBr, cm^−1^) shown in Fig. 7 ▸ exhibits the following characteristic bands: 3210 (N—H), 1761 (acet­oxy C=O), 1651 (amide C=O). Because of the inter­action of the aromatic group with the acet­oxy carbonyl moiety, the frequency of the acet­oxy C=O stretching vibration is larger compared to the normal frequency of the stretching vibrations in esters (1740 cm^−1^).

## Synthesis and crystallization   

**Figure 8:**
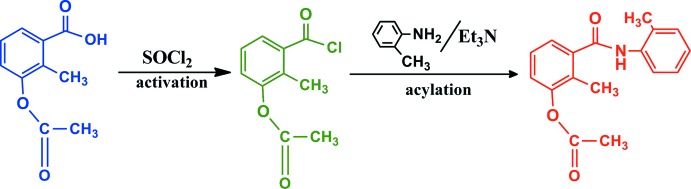
Reaction scheme.

The synthesis was performed according to the reaction scheme presented in Fig. 8 ▸ and applied earlier for the synthesis of analogous compounds (Cakmak *et al.*, 2016 ▸; Kırca *et al.*, 2018 ▸, Demir *et al.*, 2015 ▸). A solution of 3-acet­oxy-2-methyl­benzoyl chloride (11 mmol) in THF (10 mL) was added dropwise to a solution of 2-methyl­aniline (10 mmol) and tri­ethyl­amine (10 mmol) in THF (10 mL) at room temperature. After the reaction mixture had been stirred at room temperature for 15 h, the resulting white precipitate was filtered off and then 100 ml of water was added dropwise to the filtrate. The precipitate was filtered off and washed several times with water to remove the unreacted reagents and tri­ethyl­amine hydro­chloride. The crude product was recrystallized from aceto­nitrile (1.82 g, 58%; m.p. 435-438 K). Single crystals were obtained from an aceto­nitrile solution after incubation in the fridge for 20 days.

## Refinement   

Crystal data, data collection and structure refinement details are summarized in Table 3 ▸. The N-bound H atoms were freely refined. C-bound hydrogen atoms were positioned geom­etrically and refined as riding with C—H = 0.93 Å and *U*_iso_(H) = 1.2*U*_eq_(C) for aromatic C atoms and C—H = 0.96 Å and *U*_iso_(H) = 1.5*U*_eq_(C) for methyl groups. Each methyl group was allowed to rotate about its parent C—C bond.

## Supplementary Material

Crystal structure: contains datablock(s) I. DOI: 10.1107/S2056989019000021/yk2118sup1.cif

Structure factors: contains datablock(s) I. DOI: 10.1107/S2056989019000021/yk2118Isup2.hkl

Supporting information file. DOI: 10.1107/S2056989019000021/yk2118Isup3.cml

CCDC reference: 1584572

Additional supporting information:  crystallographic information; 3D view; checkCIF report

## Figures and Tables

**Figure 5:**
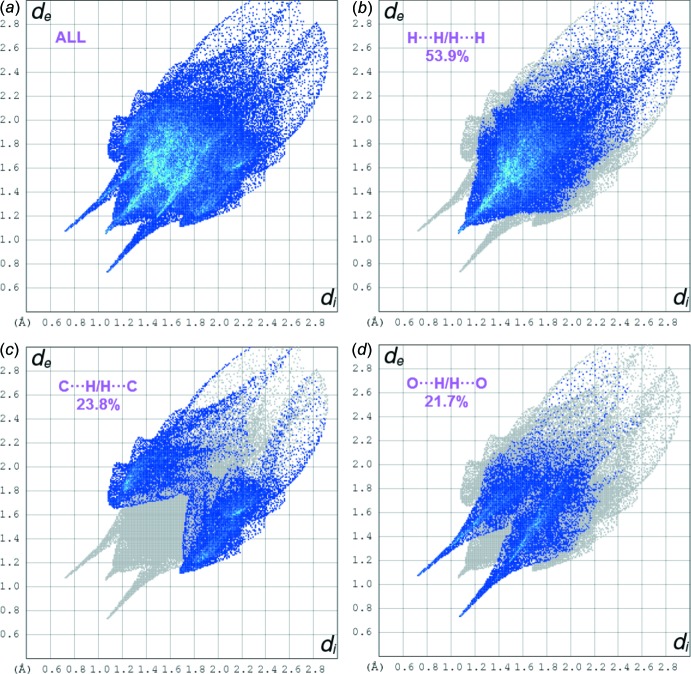
The fingerprint plots for mol­ecule *B*: (*a*) all atoms_inside_⋯all atoms_outside_ (100%), (*b*) H_inside_⋯H_outside_/H_outside_⋯H_inside_ (53.9%), (*c*) C_inside_⋯H_outside_/H_outside_⋯C_inside_ (23.8%) and (*d*) O_inside_⋯H_outside_/H_outside_⋯O_inside_ (21.7%).

**Table 1:** Selected geometric parameters (Å, °)

O1—C1	1.222 (3)	O3—C9	1.186 (4)
O4—C18	1.224 (3)	O6—C26	1.188 (4)
N1—C1	1.348 (3)	N2—C18	1.344 (4)
			
O1—C1—N1	123.5 (3)	C1—N1—C11	123.4 (2)
O4—C18—N2	123.6 (3)	C18—N2—C28	124.2 (2)
			
C9—O2—C6—C7	−100.0 (3)	C26—O5—C23—C24	−83.7 (3)
N1—C1—C2—C7	129.1 (3)	C24—C19—C18—N2	−113.6 (3)
C2—C1—N1—C11	−172.4 (2)	C28—N2—C18—C19	166.2 (2)
C1—N1—C11—C16	−66.4 (4)	C18—N2—C28—C33	66.0 (4)

**Table 2:** Hydrogen-bond geometry (Å, °) *Cg*1 is the centroid of the C28–C33 ring.

*D*—H⋯*A*	*D*—H	H⋯*A*	*D*⋯*A*	*D*—H⋯*A*
C5—H5⋯O6^i^	0.93	2.49	3.402 (4)	167
N2—H2⋯O1^ii^	0.88 (3)	1.96 (3)	2.813 (3)	164 (2)
N1—H1⋯O4	0.91 (3)	1.91 (3)	2.804 (3)	166 (2)
C25—H25*B*⋯O4	0.96	2.76	3.117 (4)	103
C34—H34*A*⋯O4	0.96	2.59	3.100 (4)	114
C8—H8*B*⋯O1	0.96	2.75	2.986 (4)	95
C3—H3⋯*Cg*1	0.93	2.81	3.666 (3)	153

Symmetry codes: (i) 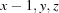; (ii) 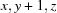.

**Table 3:** Experimental details

Crystal data	
Chemical formula	C_17_H_17_NO_3_
*M*_r_	283.31
Crystal system, space group	Triclinic, *P* 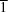
Temperature (K)	296
*a*, *b*, *c* (Å)	7.7842 (5), 8.8802 (5), 22.2112 (15)
α, β, γ (°)	94.791 (5), 97.620 (5), 90.043 (5)
*V* (Å^3^)	1516.37 (17)
*Z*	4
Radiation type	Mo *K*α
μ (mm^−1^)	0.09
Crystal size (mm)	0.42 × 0.37 × 0.21

Data collection	
Diffractometer	Stoe *IPDS* 2
Absorption correction	Integration (*X-RED32*; Stoe & Cie, 2002 ▸)
*T*_min_, *T*_max_	0.958, 0.993
No. of measured, independent and observed [*I* > 2σ(*I*)] reflections	21781, 5950, 3029
*R*_int_	0.086
(sin θ/λ)_max_ (Å^−1^)	0.617

Refinement	
*R*[*F* ^2^ > 2σ(*F* ^2^)], *wR*(*F* ^2^), *S*	0.057, 0.159, 0.90
No. of reflections	5950
No. of parameters	393
H-atom treatment	H atoms treated by a mixture of independent and constrained refinement
Δρ_max_, Δρ_min_ (e Å^−3^)	0.17, −0.14

Computer programs: *X-AREA* and *X-RED32* (Stoe & Cie, 2002 ▸), *SHELXT* (Sheldrick, 2015*a* ▸), *SHELXL2017* (Sheldrick, 2015*b* ▸), *ORTEP-3 for Windows* and *WinGX* (Farrugia, 2012 ▸), *PLATON* (Spek, 2009 ▸) and *publCIF* (Westrip, 2010 ▸).

## supplementary crystallographic information

### Crystal data

**Table:**

C_17_H_17_NO_3_	*Z* = 4
*M**_r_* = 283.31	*F*(000) = 600
Triclinic, *P*1	*D*_x_ = 1.241 Mg m^−^^3^
*a* = 7.7842 (5) Å	Mo *K*α radiation, λ = 0.71073 Å
*b* = 8.8802 (5) Å	Cell parameters from 19688 reflections
*c* = 22.2112 (15) Å	θ = 1.9–27.5°
α = 94.791 (5)°	µ = 0.09 mm^−^^1^
β = 97.620 (5)°	*T* = 296 K
γ = 90.043 (5)°	Prism, colorless
*V* = 1516.37 (17) Å^3^	0.42 × 0.37 × 0.21 mm

### Data collection

**Table:**

Stoe IPDS 2 diffractometer	5950 independent reflections
Radiation source: sealed X-ray tube, 12 x 0.4 mm long-fine focus	3029 reflections with *I* > 2σ(*I*)
Detector resolution: 6.67 pixels mm^-1^	*R*_int_ = 0.086
rotation method scans	θ_max_ = 26.0°, θ_min_ = 1.9°
Absorption correction: integration (X-RED32; Stoe & Cie, 2002)	*h* = −9→9
*T*_min_ = 0.958, *T*_max_ = 0.993	*k* = −10→10
21781 measured reflections	*l* = −27→27

### Refinement

**Table:**

Refinement on *F*^2^	0 restraints
Least-squares matrix: full	Hydrogen site location: mixed
*R*[*F*^2^ > 2σ(*F*^2^)] = 0.057	H atoms treated by a mixture of independent and constrained refinement
*wR*(*F*^2^) = 0.159	*w* = 1/[σ^2^(*F*_o_^2^) + (0.0763*P*)^2^] where *P* = (*F*_o_^2^ + 2*F*_c_^2^)/3
*S* = 0.90	(Δ/σ)_max_ < 0.001
5950 reflections	Δρ_max_ = 0.17 e Å^−^^3^
393 parameters	Δρ_min_ = −0.13 e Å^−^^3^

### Special details

**Table:**

Geometry. All esds (except the esd in the dihedral angle between two l.s. planes) are estimated using the full covariance matrix. The cell esds are taken into account individually in the estimation of esds in distances, angles and torsion angles; correlations between esds in cell parameters are only used when they are defined by crystal symmetry. An approximate (isotropic) treatment of cell esds is used for estimating esds involving l.s. planes.

### Fractional atomic coordinates and isotropic or equivalent isotropic displacement parameters (Å^2^)

**Table:**

	*x*	*y*	*z*	*U*_iso_*/*U*_eq_	
O1	0.5356 (3)	0.3278 (2)	0.28533 (9)	0.0605 (6)	
N2	0.6781 (3)	1.0533 (3)	0.32141 (11)	0.0482 (6)	
O2	0.2232 (3)	0.5083 (3)	0.07897 (10)	0.0717 (6)	
C1	0.5516 (3)	0.4634 (3)	0.28130 (12)	0.0448 (7)	
N1	0.6771 (3)	0.5500 (3)	0.31509 (11)	0.0486 (6)	
O5	0.7722 (3)	1.0738 (3)	0.07181 (9)	0.0662 (6)	
C28	0.5682 (4)	0.9934 (3)	0.36083 (12)	0.0468 (7)	
O4	0.7921 (3)	0.8326 (2)	0.28774 (10)	0.0608 (6)	
C19	0.8414 (3)	1.0503 (3)	0.23666 (12)	0.0438 (6)	
C18	0.7690 (3)	0.9681 (3)	0.28425 (13)	0.0464 (7)	
C11	0.8180 (3)	0.4903 (3)	0.35334 (12)	0.0463 (7)	
C16	0.7951 (4)	0.4184 (3)	0.40468 (13)	0.0537 (7)	
C2	0.4280 (3)	0.5450 (3)	0.23801 (13)	0.0452 (7)	
C3	0.3430 (4)	0.6702 (3)	0.26034 (15)	0.0567 (8)	
H3	0.368858	0.706558	0.301043	0.068*	
C12	0.9848 (4)	0.5130 (3)	0.33845 (14)	0.0572 (8)	
H12	0.999896	0.565609	0.304998	0.069*	
C7	0.3944 (4)	0.4892 (3)	0.17655 (14)	0.0516 (7)	
C17	0.6204 (4)	0.4057 (4)	0.42552 (15)	0.0705 (9)	
H17A	0.558650	0.319989	0.403650	0.106*	
H17B	0.555999	0.495828	0.417841	0.106*	
H17C	0.634656	0.393102	0.468398	0.106*	
C20	0.9676 (4)	1.1600 (3)	0.25396 (14)	0.0560 (8)	
H20	1.006541	1.184171	0.295057	0.067*	
C6	0.2705 (4)	0.5654 (4)	0.14029 (14)	0.0585 (8)	
C24	0.7774 (4)	1.0137 (3)	0.17529 (14)	0.0532 (7)	
C34	0.8224 (4)	0.8962 (4)	0.42816 (16)	0.0729 (9)	
H34A	0.864042	0.813155	0.403828	0.109*	
H34B	0.842987	0.876968	0.470381	0.109*	
H34C	0.882266	0.987300	0.422249	0.109*	
C23	0.8470 (4)	1.0938 (3)	0.13335 (13)	0.0545 (8)	
O6	0.9365 (4)	0.8729 (3)	0.05401 (12)	0.0885 (8)	
C33	0.6320 (4)	0.9139 (3)	0.40945 (14)	0.0550 (7)	
C13	1.1258 (4)	0.4580 (4)	0.37307 (17)	0.0728 (10)	
H13	1.236428	0.472783	0.362982	0.087*	
C22	0.9755 (4)	1.2010 (4)	0.14968 (15)	0.0639 (9)	
H22	1.020909	1.250648	0.119977	0.077*	
C26	0.8233 (5)	0.9545 (4)	0.03635 (15)	0.0646 (9)	
C4	0.2194 (4)	0.7415 (4)	0.22218 (18)	0.0727 (10)	
H4	0.161175	0.824827	0.237254	0.087*	
C32	0.5113 (5)	0.8545 (4)	0.44225 (15)	0.0713 (9)	
H32	0.550319	0.797517	0.474527	0.086*	
C29	0.3907 (4)	1.0207 (3)	0.34776 (15)	0.0608 (8)	
H29	0.350054	1.078584	0.315939	0.073*	
C5	0.1834 (4)	0.6884 (4)	0.16207 (18)	0.0736 (10)	
H5	0.100416	0.735476	0.136156	0.088*	
C15	0.9407 (4)	0.3628 (4)	0.43830 (15)	0.0678 (9)	
H15	0.928027	0.311797	0.472381	0.081*	
C21	1.0363 (4)	1.2343 (4)	0.21012 (16)	0.0680 (9)	
H21	1.123516	1.306624	0.221625	0.082*	
C9	0.2956 (5)	0.5755 (5)	0.03514 (16)	0.0724 (10)	
C31	0.3375 (5)	0.8771 (4)	0.42858 (17)	0.0758 (10)	
H31	0.260317	0.834551	0.451079	0.091*	
C14	1.1044 (5)	0.3818 (4)	0.42210 (17)	0.0739 (10)	
H14	1.200093	0.342190	0.444874	0.089*	
C25	0.6354 (5)	0.8978 (4)	0.15608 (16)	0.0806 (11)	
H25A	0.568073	0.923610	0.119042	0.121*	
H25B	0.562053	0.895667	0.187499	0.121*	
H25C	0.685593	0.800058	0.149429	0.121*	
O3	0.4029 (4)	0.6720 (3)	0.04766 (13)	0.1018 (9)	
C30	0.2768 (4)	0.9620 (4)	0.38199 (17)	0.0752 (10)	
H30	0.158693	0.979873	0.373513	0.090*	
C8	0.4864 (5)	0.3554 (4)	0.15108 (15)	0.0709 (9)	
H8A	0.432986	0.264201	0.160420	0.106*	
H8B	0.605821	0.358900	0.168815	0.106*	
H8C	0.479238	0.357183	0.107663	0.106*	
C10	0.2217 (5)	0.5106 (5)	−0.02652 (16)	0.0945 (13)	
H10A	0.118483	0.563994	−0.040154	0.142*	
H10B	0.193802	0.405789	−0.025063	0.142*	
H10C	0.304914	0.520111	−0.054284	0.142*	
C27	0.7180 (5)	0.9428 (5)	−0.02461 (16)	0.0866 (11)	
H27A	0.778041	0.882794	−0.052970	0.130*	
H27B	0.699550	1.042040	−0.038207	0.130*	
H27C	0.608231	0.896027	−0.022119	0.130*	
H2	0.653 (3)	1.146 (3)	0.3128 (11)	0.043 (7)*	
H1	0.696 (3)	0.644 (3)	0.3039 (12)	0.050 (8)*	

### Atomic displacement parameters (Å^2^)

**Table:**

	*U*^11^	*U*^22^	*U*^33^	*U*^12^	*U*^13^	*U*^23^
O1	0.0662 (13)	0.0394 (12)	0.0731 (14)	−0.0031 (10)	−0.0063 (10)	0.0143 (10)
N2	0.0520 (14)	0.0373 (13)	0.0590 (15)	0.0039 (11)	0.0162 (12)	0.0120 (12)
O2	0.0670 (14)	0.0830 (16)	0.0625 (15)	−0.0198 (12)	−0.0088 (11)	0.0188 (12)
C1	0.0410 (15)	0.0385 (16)	0.0560 (17)	−0.0008 (12)	0.0073 (13)	0.0098 (13)
N1	0.0473 (14)	0.0361 (13)	0.0616 (15)	−0.0058 (11)	−0.0006 (11)	0.0121 (12)
O5	0.0718 (14)	0.0768 (15)	0.0500 (13)	0.0150 (12)	0.0071 (11)	0.0076 (11)
C28	0.0541 (17)	0.0373 (15)	0.0503 (17)	−0.0026 (13)	0.0124 (13)	0.0025 (13)
O4	0.0714 (14)	0.0373 (11)	0.0799 (15)	0.0040 (10)	0.0271 (11)	0.0141 (10)
C19	0.0424 (15)	0.0389 (15)	0.0523 (18)	0.0006 (12)	0.0119 (13)	0.0075 (13)
C18	0.0422 (15)	0.0405 (17)	0.0565 (18)	−0.0036 (13)	0.0057 (13)	0.0064 (14)
C11	0.0478 (16)	0.0380 (15)	0.0510 (17)	−0.0028 (13)	0.0001 (13)	0.0021 (13)
C16	0.0569 (18)	0.0495 (17)	0.0538 (18)	−0.0046 (14)	0.0028 (14)	0.0064 (14)
C2	0.0387 (15)	0.0391 (15)	0.0582 (19)	−0.0023 (12)	0.0039 (13)	0.0111 (14)
C3	0.0539 (18)	0.0506 (18)	0.065 (2)	0.0044 (15)	0.0038 (15)	0.0091 (15)
C12	0.0500 (18)	0.0579 (19)	0.0633 (19)	−0.0053 (15)	0.0046 (15)	0.0080 (15)
C7	0.0455 (16)	0.0484 (17)	0.0611 (19)	−0.0051 (13)	0.0033 (14)	0.0117 (15)
C17	0.068 (2)	0.080 (2)	0.067 (2)	−0.0040 (18)	0.0158 (17)	0.0151 (18)
C20	0.0559 (18)	0.0562 (18)	0.0562 (18)	−0.0131 (15)	0.0101 (14)	0.0026 (15)
C6	0.0532 (18)	0.062 (2)	0.058 (2)	−0.0064 (16)	−0.0075 (15)	0.0158 (16)
C24	0.0503 (17)	0.0479 (17)	0.062 (2)	0.0002 (14)	0.0069 (15)	0.0078 (15)
C34	0.065 (2)	0.085 (2)	0.068 (2)	0.0039 (19)	0.0013 (17)	0.0160 (19)
C23	0.0582 (18)	0.0571 (18)	0.0504 (18)	0.0051 (15)	0.0124 (15)	0.0093 (15)
O6	0.0905 (18)	0.0892 (18)	0.0804 (17)	0.0270 (16)	−0.0028 (14)	−0.0017 (14)
C33	0.0586 (18)	0.0497 (17)	0.0571 (19)	−0.0006 (14)	0.0090 (15)	0.0047 (15)
C13	0.0478 (19)	0.084 (2)	0.085 (3)	−0.0015 (17)	0.0010 (17)	0.009 (2)
C22	0.069 (2)	0.061 (2)	0.068 (2)	−0.0093 (17)	0.0290 (17)	0.0134 (17)
C26	0.060 (2)	0.075 (2)	0.059 (2)	−0.0054 (18)	0.0066 (17)	0.0032 (19)
C4	0.060 (2)	0.067 (2)	0.090 (3)	0.0220 (17)	0.0032 (19)	0.009 (2)
C32	0.085 (3)	0.071 (2)	0.063 (2)	−0.0019 (19)	0.0214 (18)	0.0162 (18)
C29	0.0547 (19)	0.0576 (19)	0.073 (2)	0.0033 (15)	0.0166 (16)	0.0071 (16)
C5	0.056 (2)	0.071 (2)	0.091 (3)	0.0117 (18)	−0.0102 (18)	0.024 (2)
C15	0.069 (2)	0.071 (2)	0.062 (2)	0.0009 (18)	−0.0044 (17)	0.0197 (17)
C21	0.070 (2)	0.066 (2)	0.070 (2)	−0.0283 (17)	0.0181 (17)	0.0029 (18)
C9	0.059 (2)	0.084 (3)	0.074 (2)	−0.0010 (19)	−0.0024 (18)	0.022 (2)
C31	0.071 (2)	0.085 (3)	0.079 (2)	−0.004 (2)	0.034 (2)	0.014 (2)
C14	0.061 (2)	0.079 (2)	0.079 (2)	0.0102 (18)	−0.0053 (18)	0.013 (2)
C25	0.074 (2)	0.091 (3)	0.073 (2)	−0.036 (2)	−0.0032 (18)	0.013 (2)
O3	0.097 (2)	0.108 (2)	0.101 (2)	−0.0409 (18)	0.0092 (16)	0.0158 (17)
C30	0.054 (2)	0.083 (2)	0.093 (3)	0.0012 (18)	0.0241 (19)	0.009 (2)
C8	0.080 (2)	0.068 (2)	0.063 (2)	0.0065 (18)	0.0068 (17)	0.0001 (17)
C10	0.082 (3)	0.135 (4)	0.064 (2)	−0.010 (3)	−0.0021 (19)	0.020 (2)
C27	0.075 (2)	0.119 (3)	0.063 (2)	−0.005 (2)	0.0047 (18)	−0.008 (2)

### Geometric parameters (Å, º)

**Table:**

O1—C1	1.222 (3)	C24—C25	1.504 (4)
O4—C18	1.224 (3)	C34—C33	1.497 (4)
N1—C1	1.348 (3)	C34—H34A	0.9600
O3—C9	1.186 (4)	C34—H34B	0.9600
O6—C26	1.188 (4)	C34—H34C	0.9600
N2—C18	1.344 (4)	C23—C22	1.372 (4)
N2—C28	1.434 (3)	C33—C32	1.391 (4)
N2—H2	0.88 (3)	C13—C14	1.357 (5)
O2—C9	1.365 (4)	C13—H13	0.9300
O2—C6	1.414 (4)	C22—C21	1.371 (4)
C1—C2	1.499 (4)	C22—H22	0.9300
N1—C11	1.427 (3)	C26—C27	1.483 (5)
N1—H1	0.91 (3)	C4—C5	1.371 (5)
O5—C26	1.359 (4)	C4—H4	0.9300
O5—C23	1.409 (4)	C32—C31	1.365 (5)
C28—C33	1.378 (4)	C32—H32	0.9300
C28—C29	1.400 (4)	C29—C30	1.372 (4)
C19—C20	1.378 (4)	C29—H29	0.9300
C19—C24	1.399 (4)	C5—H5	0.9300
C19—C18	1.502 (4)	C15—C14	1.383 (5)
C11—C16	1.384 (4)	C15—H15	0.9300
C11—C12	1.400 (4)	C21—H21	0.9300
C16—C15	1.387 (4)	C9—C10	1.482 (5)
C16—C17	1.501 (4)	C31—C30	1.365 (5)
C2—C3	1.384 (4)	C31—H31	0.9300
C2—C7	1.404 (4)	C14—H14	0.9300
C3—C4	1.385 (4)	C25—H25A	0.9600
C3—H3	0.9300	C25—H25B	0.9600
C12—C13	1.369 (4)	C25—H25C	0.9600
C12—H12	0.9300	C30—H30	0.9300
C7—C6	1.387 (4)	C8—H8A	0.9600
C7—C8	1.497 (4)	C8—H8B	0.9600
C17—H17A	0.9600	C8—H8C	0.9600
C17—H17B	0.9600	C10—H10A	0.9600
C17—H17C	0.9600	C10—H10B	0.9600
C20—C21	1.383 (4)	C10—H10C	0.9600
C20—H20	0.9300	C27—H27A	0.9600
C6—C5	1.374 (5)	C27—H27B	0.9600
C24—C23	1.382 (4)	C27—H27C	0.9600
			
O1—C1—N1	123.5 (3)	C32—C33—C34	120.9 (3)
O4—C18—N2	123.6 (3)	C14—C13—C12	120.1 (3)
C1—N1—C11	123.4 (2)	C14—C13—H13	120.0
C18—N2—C28	124.2 (2)	C12—C13—H13	120.0
C18—N2—H2	118.5 (17)	C21—C22—C23	119.4 (3)
C28—N2—H2	113.7 (17)	C21—C22—H22	120.3
C9—O2—C6	117.7 (2)	C23—C22—H22	120.3
O1—C1—C2	121.1 (2)	O6—C26—O5	122.6 (3)
N1—C1—C2	115.4 (2)	O6—C26—C27	126.8 (4)
C1—N1—H1	118.2 (17)	O5—C26—C27	110.6 (3)
C11—N1—H1	114.9 (17)	C5—C4—C3	119.6 (3)
C26—O5—C23	118.4 (2)	C5—C4—H4	120.2
C33—C28—C29	121.0 (3)	C3—C4—H4	120.2
C33—C28—N2	122.5 (3)	C31—C32—C33	122.1 (3)
C29—C28—N2	116.5 (2)	C31—C32—H32	118.9
C20—C19—C24	121.4 (2)	C33—C32—H32	118.9
C20—C19—C18	119.9 (3)	C30—C29—C28	119.8 (3)
C24—C19—C18	118.7 (2)	C30—C29—H29	120.1
O4—C18—C19	121.1 (3)	C28—C29—H29	120.1
N2—C18—C19	115.3 (2)	C4—C5—C6	119.6 (3)
C16—C11—C12	120.3 (3)	C4—C5—H5	120.2
C16—C11—N1	122.5 (2)	C6—C5—H5	120.2
C12—C11—N1	117.1 (2)	C14—C15—C16	121.3 (3)
C11—C16—C15	117.9 (3)	C14—C15—H15	119.4
C11—C16—C17	121.8 (3)	C16—C15—H15	119.4
C15—C16—C17	120.3 (3)	C22—C21—C20	119.8 (3)
C3—C2—C7	121.3 (3)	C22—C21—H21	120.1
C3—C2—C1	119.0 (3)	C20—C21—H21	120.1
C7—C2—C1	119.6 (2)	O3—C9—O2	121.8 (3)
C4—C3—C2	120.1 (3)	O3—C9—C10	127.5 (4)
C4—C3—H3	119.9	O2—C9—C10	110.7 (3)
C2—C3—H3	119.9	C30—C31—C32	120.2 (3)
C13—C12—C11	120.1 (3)	C30—C31—H31	119.9
C13—C12—H12	119.9	C32—C31—H31	119.9
C11—C12—H12	119.9	C13—C14—C15	120.2 (3)
C6—C7—C2	116.1 (3)	C13—C14—H14	119.9
C6—C7—C8	121.5 (3)	C15—C14—H14	119.9
C2—C7—C8	122.4 (3)	C24—C25—H25A	109.5
C16—C17—H17A	109.5	C24—C25—H25B	109.5
C16—C17—H17B	109.5	H25A—C25—H25B	109.5
H17A—C17—H17B	109.5	C24—C25—H25C	109.5
C16—C17—H17C	109.5	H25A—C25—H25C	109.5
H17A—C17—H17C	109.5	H25B—C25—H25C	109.5
H17B—C17—H17C	109.5	C31—C30—C29	119.7 (3)
C19—C20—C21	119.9 (3)	C31—C30—H30	120.2
C19—C20—H20	120.0	C29—C30—H30	120.2
C21—C20—H20	120.0	C7—C8—H8A	109.5
C5—C6—C7	123.1 (3)	C7—C8—H8B	109.5
C5—C6—O2	118.2 (3)	H8A—C8—H8B	109.5
C7—C6—O2	118.6 (3)	C7—C8—H8C	109.5
C23—C24—C19	116.4 (3)	H8A—C8—H8C	109.5
C23—C24—C25	121.7 (3)	H8B—C8—H8C	109.5
C19—C24—C25	121.8 (3)	C9—C10—H10A	109.5
C33—C34—H34A	109.5	C9—C10—H10B	109.5
C33—C34—H34B	109.5	H10A—C10—H10B	109.5
H34A—C34—H34B	109.5	C9—C10—H10C	109.5
C33—C34—H34C	109.5	H10A—C10—H10C	109.5
H34A—C34—H34C	109.5	H10B—C10—H10C	109.5
H34B—C34—H34C	109.5	C26—C27—H27A	109.5
C22—C23—C24	122.9 (3)	C26—C27—H27B	109.5
C22—C23—O5	118.5 (3)	H27A—C27—H27B	109.5
C24—C23—O5	118.4 (3)	C26—C27—H27C	109.5
C28—C33—C32	117.0 (3)	H27A—C27—H27C	109.5
C28—C33—C34	122.1 (3)	H27B—C27—H27C	109.5
			
C9—O2—C6—C7	−100.0 (3)	C20—C19—C24—C23	−0.3 (4)
N1—C1—C2—C7	129.1 (3)	C18—C19—C24—C23	179.3 (3)
C2—C1—N1—C11	−172.4 (2)	C20—C19—C24—C25	−177.9 (3)
C1—N1—C11—C16	−66.4 (4)	C18—C19—C24—C25	1.7 (4)
C26—O5—C23—C24	−83.7 (3)	C19—C24—C23—C22	1.8 (4)
C24—C19—C18—N2	−113.6 (3)	C25—C24—C23—C22	179.4 (3)
C28—N2—C18—C19	166.2 (2)	C19—C24—C23—O5	−172.5 (2)
C18—N2—C28—C33	66.0 (4)	C25—C24—C23—O5	5.1 (4)
N1—C1—C2—C3	−53.9 (3)	C26—O5—C23—C22	101.7 (3)
O1—C1—N1—C11	8.5 (4)	C29—C28—C33—C32	3.9 (4)
C18—N2—C28—C29	−114.4 (3)	N2—C28—C33—C32	−176.5 (3)
C28—N2—C18—O4	−13.4 (4)	C29—C28—C33—C34	−173.8 (3)
C20—C19—C18—O4	−114.4 (3)	N2—C28—C33—C34	5.8 (4)
C24—C19—C18—O4	66.0 (4)	C11—C12—C13—C14	−0.3 (5)
C20—C19—C18—N2	66.0 (3)	C24—C23—C22—C21	−1.6 (5)
C1—N1—C11—C12	116.4 (3)	O5—C23—C22—C21	172.7 (3)
C12—C11—C16—C15	−3.6 (4)	C23—O5—C26—O6	−5.3 (5)
N1—C11—C16—C15	179.2 (3)	C23—O5—C26—C27	174.0 (3)
C12—C11—C16—C17	173.8 (3)	C2—C3—C4—C5	−0.8 (5)
N1—C11—C16—C17	−3.3 (4)	C28—C33—C32—C31	−2.0 (5)
O1—C1—C2—C3	125.3 (3)	C34—C33—C32—C31	175.7 (3)
O1—C1—C2—C7	−51.7 (4)	C33—C28—C29—C30	−3.0 (5)
C7—C2—C3—C4	1.3 (4)	N2—C28—C29—C30	177.4 (3)
C1—C2—C3—C4	−175.6 (3)	C3—C4—C5—C6	−0.1 (5)
C16—C11—C12—C13	3.0 (4)	C7—C6—C5—C4	0.6 (5)
N1—C11—C12—C13	−179.7 (3)	O2—C6—C5—C4	176.4 (3)
C3—C2—C7—C6	−0.8 (4)	C11—C16—C15—C14	1.6 (5)
C1—C2—C7—C6	176.1 (2)	C17—C16—C15—C14	−175.9 (3)
C3—C2—C7—C8	179.1 (3)	C23—C22—C21—C20	−0.1 (5)
C1—C2—C7—C8	−4.0 (4)	C19—C20—C21—C22	1.6 (5)
C24—C19—C20—C21	−1.4 (4)	C6—O2—C9—O3	4.8 (5)
C18—C19—C20—C21	179.0 (3)	C6—O2—C9—C10	−175.4 (3)
C2—C7—C6—C5	−0.1 (4)	C33—C32—C31—C30	−0.9 (6)
C8—C7—C6—C5	179.9 (3)	C12—C13—C14—C15	−1.7 (5)
C2—C7—C6—O2	−175.9 (2)	C16—C15—C14—C13	1.0 (5)
C8—C7—C6—O2	4.1 (4)	C32—C31—C30—C29	2.0 (6)
C9—O2—C6—C5	84.0 (4)	C28—C29—C30—C31	−0.1 (5)

### Hydrogen-bond geometry (Å, º)

*Cg*1 is the centroid of the C28–C33 ring.

**Table:**

*D*—H···*A*	*D*—H	H···*A*	*D*···*A*	*D*—H···*A*
C5—H5···O6^i^	0.93	2.49	3.402 (4)	167
N2—H2···O1^ii^	0.88 (3)	1.96 (3)	2.813 (3)	164 (2)
N1—H1···O4	0.91 (3)	1.91 (3)	2.804 (3)	166 (2)
C25—H25*B*···O4	0.96	2.76	3.117 (4)	103
C34—H34*A*···O4	0.96	2.59	3.100 (4)	114
C8—H8*B*···O1	0.96	2.75	2.986 (4)	95
C3—H3···*Cg*1	0.93	2.81	3.666 (3)	153

Symmetry codes: (i) *x*−1, *y*, *z*; (ii) *x*, *y*+1, *z*.
